# Dendritic Cells as Immunometabolic Regulatory Nodes in Diabetes: Molecular Mechanisms and Therapeutic Reprogramming

**DOI:** 10.3390/ijms27136057

**Published:** 2026-07-06

**Authors:** Fangfang Jin, Weidong Wu, Xuan Yang, Xiang Fan, Xiaosen Zhao, Wei Liu, Xinrong Fan

**Affiliations:** 1 Experimental Research Center, China Academy of Chinese Medical Sciences, Beijing 100700, China; jinfangfang99@163.com (F.J.); m13176218395@163.com (X.Y.); zxs_99@163.com (X.Z.); 2 School of Traditional Chinese Medicine, Shaanxi University of Chinese Medicine, Xianyang 712046, China; wuwd0116@163.com; 3 School of Traditional Chinese Medicine, Beijing University of Chinese Medicine, Beijing 100029, China; xiang202208@163.com

**Keywords:** dendritic cells, type 1 diabetes mellitus, type 2 diabetes mellitus, immunometabolism, metabolic inflammation

## Abstract

Diabetes mellitus comprises a group of heterogeneous metabolic disorders characterized by persistent hyperglycemia, progressive β-cell dysfunction, and multi-organ complications. Although type 1 diabetes mellitus (T1DM) and type 2 diabetes mellitus (T2DM) have distinct pathogenic origins, both involve immune dysregulation, tissue stress, release of danger signals, and chronic inflammation. Dendritic cells (DCs), as antigen-presenting cells, integrate innate immune sensing, antigen presentation, cytokine production, T-cell regulation, and peripheral immune tolerance, placing them at a critical interface between autoimmunity and metabolic inflammation. In T1DM, DCs contribute to β-cell autoantigen presentation, tolerance breakdown, autoreactive T-cell activation, and insulitis amplification. In T2DM, DCs may contribute to adipose tissue inflammation, hepatic immunometabolic crosstalk, β-cell stress, vascular inflammation, and immune remodeling associated with diabetes-related complications. Here, we review the disease-specific roles of DC subsets in T1DM and T2DM and discuss shared molecular mechanisms, including pattern-recognition receptor signaling, metabolic reprogramming, inflammasome activation, cytokine networks, and the shift from immune tolerance to inflammation. We also evaluate therapeutic DC reprogramming strategies and their potential implications for targeted immunometabolic intervention in diabetes.

## 1. Introduction

Diabetes mellitus comprises a heterogeneous group of chronic metabolic diseases characterized by persistent hyperglycemia, progressive β-cell dysfunction, and long-term multi-organ complications. Traditionally, type 1 diabetes mellitus (T1DM) and type 2 diabetes mellitus (T2DM) have been regarded as relatively distinct pathogenic entities: T1DM is primarily driven by autoimmune destruction of pancreatic β-cells, whereas T2DM develops in the context of obesity, insulin resistance, ectopic lipid accumulation, metabolic inflammation, and β-cell stress [1,2,3]. However, increasing evidence indicates that diabetes-associated immune dysregulation is not confined to classical autoimmunity. β-cell injury, tissue stress, and metabolic overload can translate metabolic disturbance into persistent immune responses by inducing the release of danger signals, innate immune activation, and chronic low-grade inflammation, thereby contributing to the progression of different forms of diabetes [4,5,6].

Dendritic cells (DCs) occupy a pivotal position at the interface between innate immune sensing and adaptive immune regulation [7]. As antigen-presenting cells, DCs can capture and process antigens, sense pathogen-associated molecular patterns (PAMPs) and damage-associated molecular patterns (DAMPs), secrete cytokines, regulate T-cell priming, and maintain peripheral immune tolerance [8,9,10]. These functions position DCs as important regulators of diabetic immunopathology. In T1DM, DCs can present β-cell autoantigens, promote autoreactive T-cell activation, and amplify insulitis [11,12]. In T2DM, DCs are exposed to high glucose, free fatty acids, oxidized lipids, advanced glycation end products, adipokines, and inflammatory mediators, and may participate in adipose tissue inflammation, hepatic immunometabolic crosstalk, vascular inflammation, β-cell stress, and diabetes-related complications [13,14,15]. Thus, DCs do not operate through a single disease mechanism; rather, their functions depend on the metabolic state and inflammatory microenvironment of the tissue in which they reside.

Although the role of DCs in diabetic immunopathology has attracted increasing attention, the existing literature remains fragmented. Previous reviews on DCs in diabetes have largely focused on the autoimmune context of T1DM, particularly β-cell autoantigen presentation, tolerance breakdown, and autoreactive T-cell priming. In contrast, evidence regarding DCs in T2DM remains largely fragmented, scattered across studies on obesity-related metabolic inflammation, adipose tissue immune remodeling, insulin resistance, vascular inflammation, and diabetes-associated complications, with limited systematic integration. More importantly, there remains a lack of a framework that simultaneously compares the disease-specific roles of DCs in T1DM and T2DM while identifying their shared immunometabolic mechanisms. In addition, the tissue localization, functional specialization, and stage-dependent roles of distinct DC subsets in both diseases are not fully defined, and the preclinical evidence, early clinical findings, and translational boundaries of DC-related interventions require systematic evaluation.

To address these issues, this review places DCs within an integrated framework of immunometabolic regulation in diabetes. We first compare the disease-specific roles of DCs in T1DM and T2DM, with emphasis on β-cell antigen presentation, tolerance breakdown, metabolic inflammation, and tissue immune remodeling. We then define the shared molecular regulatory mechanisms through which DCs may operate in both forms of diabetes, including danger signal sensing, metabolic reprogramming, inflammasome activation, cytokine networks, and the shift from immune tolerance to inflammation. Finally, we evaluate the therapeutic potential of reprogramming DC molecular states and the associated challenges for clinical translation. By integrating disease-specific mechanisms with shared immunometabolic pathways, this review aims to establish a clearer, more integrative, and translationally relevant framework that may provide a theoretical basis for precision immunometabolic intervention in diabetes.

## 2. Disease-Specific Roles of DCs in T1DM and T2DM

**Figure 1:**
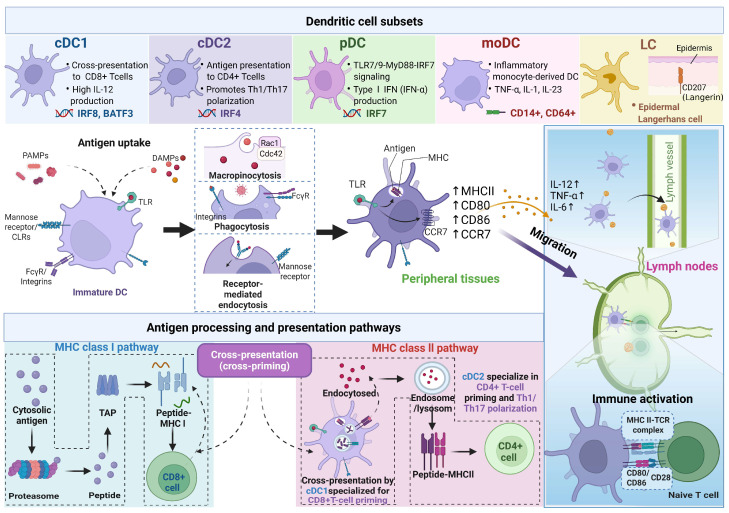
Functional overview of dendritic cell subsets, maturation, antigen presentation, and T-cell activation. The schematic summarizes the heterogeneity and core immunological functions of dendritic cells (DCs). The upper panel shows major DC subsets, including conventional type 1 DCs (cDC1s), conventional type 2 DCs (cDC2s), plasmacytoid DCs (pDCs), monocyte-derived DCs (moDCs), and Langerhans cells (LCs), together with representative functional features and key transcriptional regulators. In peripheral tissues, immature DCs recognize pathogen-associated molecular patterns and damage-associated molecular patterns through pattern-recognition and uptake receptors, including Toll-like receptors, mannose receptor/C-type lectin receptors, Fcγ receptors, and integrin-associated pathways. Antigen uptake occurs through macropinocytosis, phagocytosis, and receptor-mediated endocytosis. After activation, DCs undergo maturation, characterized by antigen processing, increased expression of MHC II, CD80, CD86, and CCR7, and migration through afferent lymphatic vessels to draining lymph nodes. Mature DCs present antigens to naïve T cells through MHC–TCR interactions and provide costimulatory signals via CD80/CD86–CD28, thereby promoting T-cell activation. The lower panel illustrates MHC class I, MHC class II, and cross-presentation pathways, highlighting the specialization of cDC1s in cross-presentation to CD8^+^ T cells and cDC2s in antigen presentation to CD4^+^ T cells. Abbreviations: BATF3, basic leucine zipper ATF-like transcription factor 3; Cdc42, cell division control protein 42 homolog; CD, cluster of differentiation; cDC1, conventional type 1 dendritic cell; cDC2, conventional type 2 dendritic cell; CCR7, C-C chemokine receptor 7; CLRs, C-type lectin receptors; DAMPs, damage-associated molecular patterns; DCs, dendritic cells; FcγR, Fc gamma receptor; IFN, interferon; IL, interleukin; IRF, interferon regulatory factor; LC, Langerhans cell; MHC, major histocompatibility complex; moDC, monocyte-derived dendritic cell; MyD88, myeloid differentiation primary response 88; PAMPs, pathogen-associated molecular patterns; pDC, plasmacytoid dendritic cell; Rac1, Ras-related C3 botulinum toxin substrate 1; TAP, transporter associated with antigen processing; TCR, T-cell receptor; Th1, T helper 1 cell; Th17, T helper 17 cell; TLR, Toll-like receptor; TNF-α, tumor necrosis factor-α. Created in BioRender. Jin, F. (2026) https://BioRender.com/fsr3tn3.

DCs are antigen-presenting cells uniquely equipped to integrate antigen-derived, inflammatory, and metabolic cues. Through antigen uptake, processing, and presentation, pattern-recognition receptor signaling, cytokine production, co-stimulatory molecule expression, T-cell priming, and maintenance of peripheral tolerance, DCs shape whether tissue-derived signals induce tolerogenic or immunogenic responses [16,17,18]. This functional plasticity is underpinned by DC subset heterogeneity. According to developmental origin and functional specialization, DCs can be broadly classified into conventional type 1 DCs (cDC1s), conventional type 2 DCs (cDC2s), plasmacytoid DCs (pDCs), monocyte-derived DCs (moDCs), and Langerhans cells (LCs) [19]. cDC1s are specialized for cross-presentation and CD8^+^ T-cell activation; cDC2s preferentially regulate CD4^+^ T-cell differentiation; pDCs are characterized by nucleic acid sensing and type I interferon (IFN-I) production; moDCs commonly arise under inflammatory conditions and amplify local immune responses; and LCs mainly contribute to immune surveillance in barrier tissues. These differences in antigen-presenting capacity, cytokine profile, tissue localization, and regulatory potential provide the cellular basis for the context-dependent and disease-specific roles of DCs in T1DM and T2DM. The major DC subsets and their core functions in antigen uptake, maturation, antigen presentation, and T-cell activation are summarized in Figure 1. The developmental trajectory and transcriptional regulation of cDC1 and cDC2 lineages, including the roles of IRF8, BATF3, IRF4, KLF4, and Notch2, are further illustrated in Supplementary Figure S1.

### 2.1. DCs in T1DM: β-Cell Antigen Presentation, Tolerance Breakdown, and Autoimmune Amplification

T1DM is an autoimmune disease targeting pancreatic β-cells. Genetic susceptibility and environmental factors can promote the breakdown of immune tolerance, while DCs provide a cellular link between β-cell injury, innate danger signal sensing, and autoreactive T-cell priming [20,21,22,23]. Therefore, during the immunopathological process of T1DM, DCs are not only presenters of β-cell antigens but also key regulators of autoimmune initiation, effector T-cell polarization, and the persistence of islet inflammation. To strengthen this disease-specific framework, DC involvement in T1DM can be understood as a stage-dependent process that links β-cell stress, antigen acquisition, innate danger sensing, loss of peripheral tolerance, autoreactive T-cell priming, islet re-entry of effector cells, epitope spreading, and autoimmune memory maintenance.

DC-related immune responses in T1DM may be driven by autoantigens and danger signals released from stressed or dying β-cells [24]. β-cell antigens such as insulin, glutamic acid decarboxylase 65 (GAD65), and insulinoma-associated antigen-2 (IA-2) can be taken up by local islet-resident or migratory DCs [24]. Under steady-state conditions, immature DCs can present self-antigens with limited co-stimulation, thereby contributing to the maintenance of peripheral immune tolerance. However, in hosts with T1DM-related genetic susceptibility, particularly under local inflammatory conditions, DCs may shift from a tolerogenic state to an immunogenic state. Studies in non-obese diabetic (NOD) mice, a spontaneous autoimmune mouse model of T1DM, have shown that DCs can appear in the islets during early stages of islet autoimmunity, supporting their potential involvement in β-cell antigen uptake, danger signal sensing, and early autoimmune activation [25].

β-cell injury and environmental stimuli can further promote DC activation. Virus-associated nucleic acids may be sensed by DCs through pattern-recognition receptors, including Toll-like receptor 3 (TLR3) and TLR7/9, whereas TLR4 may participate in virus- or injury-associated inflammatory signaling [26,27]. TLR3 signals mainly through the Toll/interleukin-1 receptor domain-containing adaptor-inducing interferon-β (TRIF) pathway, TLR7/9 rely predominantly on myeloid differentiation primary response 88 (MyD88), and TLR4 engages both MyD88- and TRIF-related pathways. These signals activate downstream networks such as nuclear factor-κB (NF-κB), interferon regulatory factors (IRFs), and mitogen-activated protein kinase (MAPK), thereby promoting DC maturation and inflammatory cytokine expression [28]. In parallel, DAMP-related signals, including extracellular adenosine triphosphate (ATP), heat shock protein 70 (HSP70), high-mobility group box 1 (HMGB1), and β-cell apoptotic or secondary necrotic material, can further enhance DC activation or antigen presentation through P2X purinoceptor 7 (P2X7), receptor for advanced glycation end products (RAGE), scavenger receptors, TLR-related pathways, or inflammasome-dependent mechanisms [29,30,31,32,33,34]. pDCs are particularly relevant at this early danger-sensing stage. Self-nucleic acids released by dying or stressed β-cells can activate pDCs through TLR9 and induce IFN-I production, potentially enhancing β-cell major histocompatibility complex class I (MHC I) expression and susceptibility to immune recognition [35,36]. This pathway is supported by evidence that β-cell death-associated self-DNA complexed with antimicrobial peptides, such as CRAMP in NOD mice or LL37 in humans, can activate pDCs and monocytes, increase IFN-α production, and enhance antigen-presenting or co-stimulatory features, thereby linking β-cell injury to early innate immune activation in T1DM [37,38]. This process may contribute to the formation of an early autoimmune environment, although the role of pDCs may vary according to disease stage and local microenvironment.

As DCs acquire an immunogenic phenotype, their capacity for antigen presentation and T-cell priming increases. Mature DCs upregulate MHC molecules, CD80, CD86, and CD40, and produce higher levels of inflammatory mediators such as interleukin-12 (IL-12), tumor necrosis factor-α (TNF-α), and IL-6, while their tolerogenic capacity becomes diminished [17]. Activated DCs then migrate to pancreas-draining lymph nodes in a C-C chemokine receptor 7 (CCR7)-dependent manner and present β-cell antigens to autoreactive T cells [39]. This process depends on three classes of signals: recognition of peptide-MHC complexes by the T-cell receptor (TCR), co-stimulatory signaling such as CD80/CD86–CD28 interactions, and T-cell polarization mediated by DC-derived cytokines [39]. IL-12 secreted by DCs promotes T helper 1 (Th1) differentiation and IFN-γ production, whereas IL-6, IL-1β, and IL-23 can contribute to the induction, expansion, or maintenance of Th17 responses [40]. This T-cell-priming process is further shaped by DC subset specialization. In mice, cDC1s are commonly characterized by XCR1 and CLEC9A expression, with CD8α expression in lymphoid tissues or CD103 expression in peripheral tissues, and their development is closely associated with BATF3 and IRF8 [41,42,43]. Functionally, cDC1s are specialized for cross-presentation of cell-associated antigens to CD8^+^ T cells; in T1DM, this property may allow β-cell antigens to be routed into the MHC I pathway and promote cytotoxic CD8^+^ T-cell priming [44,45]. By contrast, cDC2s are commonly characterized by CD11b and SIRPα/CD172a expression and are closely associated with IRF4-related differentiation programs. They preferentially support MHC II-restricted CD4^+^ T-cell responses, contributing either to the maintenance of peripheral tolerance or, under inflammatory conditions, to pathogenic CD4^+^ T-cell activation [41,42].

Autoreactive Th1 and Th17 cells, as well as CD8^+^ cytotoxic T cells primed and polarized by DCs, can migrate back to the islet microenvironment and participate in inflammatory β-cell injury and cytotoxic destruction of β-cells. β-cell damage further releases HMGB1, uric acid crystals, and other DAMPs, which can reactivate local DCs and promote effector T-cell polarization and pro-inflammatory cytokine production, thereby amplifying islet inflammation [45,46]. At this stage, moDCs may be more prominently involved in inflammatory amplification and local tissue injury. Neutrophil extracellular traps (NETs) from patients with T1DM can induce moDC maturation, increase the expression of interferon-related inflammatory mediators, and promote glycolytic metabolic reprogramming. These moDCs can further drive CD4^+^ and CD8^+^ T cells toward IFN-γ-producing pathogenic phenotypes [47]. This NET–moDC–T-cell axis provides a mechanistic link between innate inflammatory products, DC metabolic activation, Th1-skewed immunity, and sustained β-cell-directed autoimmunity. In addition, DCs can upregulate chemokines such as C-C motif chemokine ligand 2 (CCL2) and C-X-C motif chemokine ligand 10 (CXCL10), thereby recruiting monocytes, autoreactive T cells, and other inflammatory cells to the islet microenvironment and sustaining local inflammatory infiltration. Interactions between DCs and natural killer (NK)/natural killer T (NKT) cells may further intensify local inflammation. DC-derived IL-12 and chemokines can promote NK/NKT-cell activation and tissue migration, whereas IFN-γ produced by NK cells can further promote DC maturation, forming a self-amplifying inflammatory loop [39,48,49]. Moreover, DCs may participate in the long-term maintenance of autoimmune responses. Persistently released β-cell antigens can be repeatedly captured and presented by DCs, allowing immune responses to extend from initial antigens to additional β-cell-related epitopes, a process known as epitope spreading. Molecules expressed by DCs, such as CD40 and OX40 ligand (OX40L), together with IL-15, may support the generation and maintenance of autoreactive memory T cells, allowing immune attack to persist even after the initial trigger has weakened [39]. Thus, DCs may contribute not only to the initiation of β-cell autoimmunity but also to its chronicity by repeatedly presenting newly released β-cell antigens, sustaining pathogenic T-cell help, and maintaining autoreactive memory responses.

**Figure 2:**
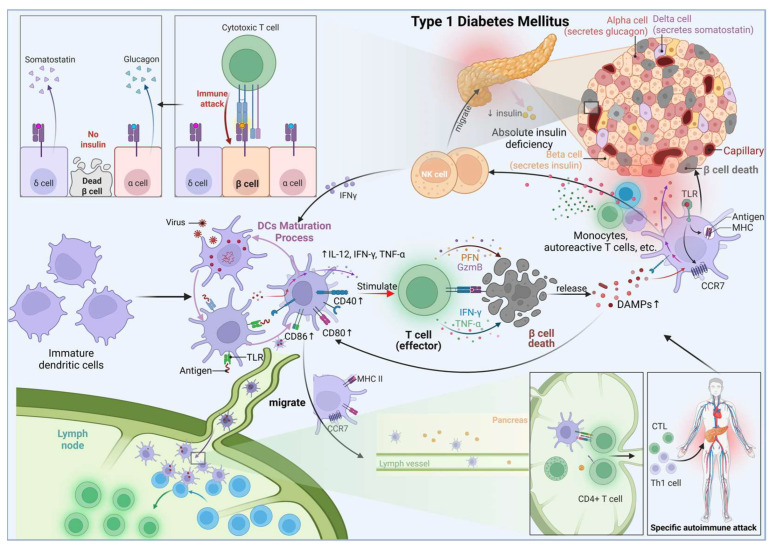
DC-mediated β-cell antigen presentation, autoreactive T-cell priming, and inflammatory amplification in type 1 diabetes mellitus. This schematic illustrates how dendritic cells (DCs) connect β-cell injury with autoimmune activation and inflammatory amplification in type 1 diabetes mellitus (T1DM). In the islet microenvironment, β-cell stress or death releases β-cell autoantigens and damage-associated molecular patterns (DAMPs), while environmental stimuli such as viral signals may further activate immature DCs through pattern-recognition receptors, including Toll-like receptors (TLRs). Activated DCs undergo maturation, upregulate co-stimulatory molecules such as CD80, CD86, and CD40, and contribute to an inflammatory milieu involving IL-12, IFN-γ, and TNF-α. Mature DCs migrate through lymphatic vessels to pancreatic draining lymph nodes in a CCR7-dependent manner, where they present β-cell antigen-derived peptides through MHC molecules and prime autoreactive T cells. This process promotes CD4^+^ T-cell/Th1 responses and CD8^+^ cytotoxic T lymphocyte responses. Activated effector T cells then return to the pancreatic islet microenvironment and mediate β-cell injury through inflammatory cytokines and cytotoxic mediators, including IFN-γ, TNF-α, perforin, and granzyme B. The upper-left panels and the upper-right islet panel illustrate the downstream consequence of this cytotoxic immune attack: selective β-cell death leads to loss of insulin secretion and progression toward absolute insulin deficiency, whereas neighboring α- and δ-cells remain relatively preserved and continue to secrete glucagon and somatostatin. Recurrent β-cell death releases additional antigens and DAMPs, which further activate local or recruited DCs, promote the recruitment of monocytes, autoreactive T cells, and NK cells, and sustain a feedback loop of insulitis, epitope spreading, and β-cell-directed autoimmune attack. Abbreviations: CCR7, C-C chemokine receptor 7; CD, cluster of differentiation; CTL, cytotoxic T lymphocyte; DAMPs, damage-associated molecular patterns; DCs, dendritic cells; GzmB, granzyme B; IFN-γ, interferon-γ; IL, interleukin; MHC, major histocompatibility complex; NK, natural killer; PFN, perforin; T1DM, type 1 diabetes mellitus; Th1, T helper 1 cell; TLRs, Toll-like receptors; TNF-α, tumor necrosis factor-α. Created in BioRender. Jin, F. (2026) https://BioRender.com/tm0xsze.

Overall, DCs participate in the breakdown of tolerance and the amplification of islet inflammation in T1DM through β-cell antigen uptake and presentation, the sensing of PAMPs and DAMPs, enhanced co-stimulation, inflammatory cytokine production, cross-priming, epitope spreading, and memory T-cell maintenance. pDCs can influence the early immune environment through nucleic acid sensing and IFN-I responses and may display compensatory regulatory properties at certain disease stages. cDC1s preferentially mediate β-cell antigen cross-presentation and CD8^+^ T-cell priming; cDC2s mainly regulate CD4^+^ T-cell tolerance and effector differentiation; and moDCs are more prominently involved in inflammatory amplification and local tissue injury. Current evidence points to a role for DCs as important regulators within the immunopathological network of T1DM, particularly during autoimmune initiation and sustained inflammation. However, the contributions of different DC subsets are not equivalent, and many proposed mechanisms remain largely based on in vivo models and in vitro studies. Future studies should integrate human islet samples, longitudinal cohorts, single-cell omics, and subset-specific functional analyses to clarify the precise roles of DCs at different stages of human T1DM. Figure 2 provides a schematic overview of DC-mediated β-cell antigen presentation, autoreactive T-cell priming, and inflammatory amplification during T1DM-associated insulitis.

### 2.2. DCs in T2DM: Metabolic Inflammation, Tissue Immune Remodeling, and β-Cell Stress

The development and progression of T2DM are jointly driven by adipocyte dysfunction, ectopic lipid accumulation, insulin resistance, macrophage-driven chronic low-grade inflammation, gut microbiota alterations, and β-cell stress [50]. In this context, DCs are better understood as context-dependent immunometabolic modulators in metabolic inflammation and tissue remodeling rather than as sole drivers of disease pathogenesis. Under conditions of obesity, a high-fat diet, hyperglycemia, and persistent metabolic stress, DCs can sense free fatty acids, oxidized lipids, advanced glycation end products (AGEs), adipokines, and inflammatory cytokines. By altering antigen presentation, co-stimulatory signaling, cytokine production, T-cell polarization, and tissue immune interactions, DCs may contribute to immunometabolic processes associated with insulin resistance, β-cell stress, and diabetes-related complications.

Adipose tissue is an important site where DCs participate in T2DM-related immunometabolic abnormalities. Under physiological conditions, adipose tissue DCs exist in a largely quiescent state and help maintain basal immune surveillance and local homeostasis. In obesity, both the abundance and composition of DCs in visceral adipose tissue are altered. Free fatty acids in a high-fat environment can also directly influence DC function [51,52]. For example, palmitate can promote DC maturation through TLR-related pathways and NF-κB signaling, upregulate CD80, CD86, and MHC II, and enhance the production of inflammatory cytokines such as IL-6 and IL-12 [53]. These changes can promote the skewing of CD4^+^ T cells toward Th1/Th17 responses and may weaken regulatory T cell (Treg)-mediated immune regulation [53]. Subsequently, IFN-γ derived from Th1 cells can promote inflammatory macrophage polarization, whereas IL-17 derived from Th17 cells may contribute to inflammatory infiltration in metabolic organs such as adipose tissue and liver [54,55,56]. Thus, DCs may participate in adipose tissue immune remodeling by shaping T-cell–macrophage interactions, thereby indirectly affecting insulin sensitivity.

In addition to free fatty acids, obesity-associated adipokines may further modulate DC function. Leptin can affect DC activation through leptin receptor b (LepRb), Janus kinase–signal transducer and activator of transcription (JAK–STAT), NF-κB, and related signaling pathways, and can enhance CCR7-associated migratory capacity [57]. Visfatin and chemerin have also been reported to participate in local inflammatory responses by promoting IL-6 production or inducing DC chemotaxis [58]. Together, these signals shape an adipose microenvironment that regulates DC function in obesity.

The liver is another important immunometabolic organ. Lipid overload, gut-derived inflammatory signals, and local cytokines can alter the abundance and activation state of hepatic DCs. Some studies suggest that hepatic DC recruitment may involve C-type lectin-dependent recognition and interactions with liver-resident cells such as Kupffer cells [59]. In a metabolic inflammatory environment, DCs may cooperate with Kupffer cells, macrophages, hepatic stellate cells, and NK cells to form a cytokine network and promote the production of inflammatory mediators such as TNF-α, IL-1β, and IL-6. These signals may affect hepatocyte insulin signaling and gluconeogenic regulation, thereby contributing to hepatic insulin resistance and increased glucose output [59,60].

Chronic metabolic stress can also directly alter DC sensing and antigen-presenting functions through oxidized lipids, AGEs, and glycoxidation products. Oxidized low-density lipoprotein (oxLDL), AGE-modified proteins, and other metabolic stress products can activate DCs through receptors such as CD36, lectin-like oxidized low-density lipoprotein receptor-1 (LOX-1), and RAGE, and can further activate NF-κB, PKCβ1, TLR4-related signaling, and other downstream inflammatory pathways. These changes enhance the expression of co-stimulatory molecules, adhesion molecules, chemokines, and inflammatory cytokines [61,62,63,64]. In addition, metabolic stress may alter the antigen-presenting profile of DCs, for example, by enhancing presentation of metabolic antigens such as apolipoprotein B (APOB) or by altering antigen presentation profiles through MHC II glycosylation/glycation-related changes [65]. These changes may promote inflammatory CD4^+^ T-cell responses and contribute to the maintenance of inflammation in metabolic tissues such as adipose tissue, the liver, and the vasculature.

DCs may also influence β-cell functional decline by sustaining an inflammatory microenvironment. Inflammatory cytokines such as IL-1β and TNF-α have been shown to induce β-cell dysfunction, endoplasmic reticulum stress, and cellular injury [66,67]. Under glucotoxic, lipotoxic, and inflammatory stress, injured β-cells can release DAMPs and β-cell-derived antigens, further activating local antigen-presenting cells, including DCs, and engaging in a self-perpetuating inflammatory loop. In parallel, DC-derived IL-6 and related factors may inhibit Treg generation or function and weaken local immune regulation [68,69]. However, this process occurs within a broader pathological network driven by lipotoxicity, glucotoxicity, macrophage-mediated inflammation, and intrinsic β-cell stress. Therefore, DCs are better regarded as contributors to β-cell stress and inflammatory maintenance rather than as the sole cause of β-cell decompensation.

Distinct DC subsets exhibit distinct functional features in T2DM and its complications. Some studies suggest that cDC1s may participate in metabolic protection in specific adipose tissue environments by regulating invariant natural killer T (iNKT) cells and macrophage responses, whereas cDC2s may contribute to the metabolic benefits of dietary fructooligosaccharides in high-fat diet models by maintaining intestinal Th17-cell homeostasis [70,71]. By contrast, pDCs can accumulate in adipose tissue or the liver during obesity and promote the recruitment of inflammatory macrophages and chronic low-grade inflammation through IFN-I/IFNAR signaling, thereby contributing to insulin resistance [72]. Experimental models also suggest that pDCs may participate in diabetes-associated vascular endothelial dysfunction by affecting endothelial nitric oxide synthase (eNOS) phosphorylation and secreting IFN-I and TNF-α [73]. moDCs are more prone to pro-inflammatory remodeling in high-glucose and oxidized lipid-rich environments. In vitro studies have shown that elevated glucose concentrations promote moDC maturation, as reflected by increased CD83 and CD86 expression and enhanced IL-6 and IL-12 secretion. In individuals with obesity and T2DM, moDCs may also display increased adhesion molecule expression and a pro-inflammatory phenotype, suggesting a potential contribution to early vascular inflammation [13,74]. Evidence for LCs in T2DM has mainly come from studies of diabetic complications involving the skin, peripheral nerves, and wound healing, rather than from studies addressing the central mechanisms underlying systemic insulin resistance [75,76]. Thus, the roles of different DC subsets in T2DM should be interpreted in relation to tissue site, disease stage, and experimental model, rather than being uniformly classified as pro-inflammatory or protective.

**Figure 3:**
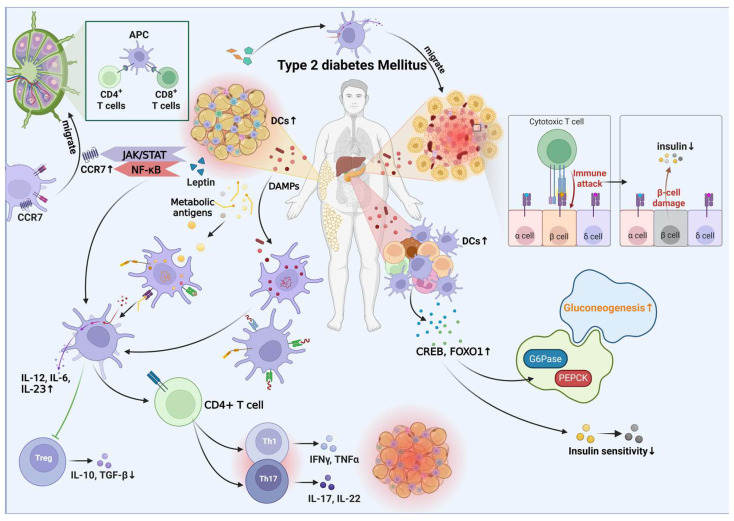
DC involvement in type 2 diabetes mellitus-associated metabolic inflammation, tissue immune remodeling, and β-cell stress. This schematic illustrates the context-dependent roles of DCs in type 2 diabetes mellitus (T2DM)-associated immunometabolic remodeling. In obesity, insulin resistance, and persistent metabolic stress, DCs in metabolic tissues such as adipose tissue and the liver can sense free fatty acids, oxidized lipids, advanced glycation end products (AGEs), adipokines, inflammatory cytokines, and gut-derived inflammatory signals. These stimuli can promote DC maturation, cytokine production, and altered antigen-presenting activity. In adipose tissue, DCs may shape T-cell–macrophage interactions by promoting Th1/Th17-skewed responses and influencing inflammatory macrophage polarization, thereby contributing to local inflammation and impaired insulin sensitivity. In the liver, DCs may interact with Kupffer cells, macrophages, hepatic stellate cells, and NK cells to participate in hepatic inflammatory networks and immune-metabolic dysregulation. In the islet microenvironment, DCs may contribute to inflammatory feedback loops associated with β-cell stress and functional decline, but they should be interpreted as participants in a broader pathological network involving glucotoxicity, lipotoxicity, macrophage-mediated inflammation, and intrinsic β-cell stress rather than as sole drivers of β-cell failure. DC subset-specific effects, including pDC–IFN-I signaling, moDC pro-inflammatory remodeling, and context-dependent cDC functions, may further modulate T2DM-associated inflammation and complications. Abbreviations: AGEs, advanced glycation end products; APC, antigen-presenting cell; cDC, conventional dendritic cell; CCR7, C-C chemokine receptor 7; CD, cluster of differentiation; CREB, cAMP response element-binding protein; DAMPs, damage-associated molecular patterns; DCs, dendritic cells; FOXO1, forkhead box O1; G6Pase, glucose-6-phosphatase; IFN-I, type I interferon; IFN-γ, interferon-γ; IL, interleukin; JAK/STAT, Janus kinase/signal transducer and activator of transcription; MHC, major histocompatibility complex; moDC, monocyte-derived dendritic cell; NF-κB, nuclear factor-κB; NK, natural killer; pDC, plasmacytoid dendritic cell; PEPCK, phosphoenolpyruvate carboxykinase; T2DM, type 2 diabetes mellitus; TGF-β, transforming growth factor-β; Th1/Th17, T helper 1/T helper 17 cells; TNF-α, tumor necrosis factor-α; Treg, regulatory T cell. Created in BioRender. Jin, F. (2026) https://BioRender.com/n7w6m8t.

Overall, the role of DCs in T2DM is not limited to inflammatory cytokine release but extends to broader immunometabolic remodeling. DCs may influence insulin sensitivity, tissue inflammation, and disease progression through adipose tissue T-cell–macrophage interactions, hepatic immunometabolic crosstalk, metabolic antigen presentation, β-cell stress feedback, and regulation of vascular endothelial function. Compared with T1DM, evidence regarding DCs in T2DM remains more heterogeneous, with many conclusions derived from animal models, in vitro cell systems, or studies of specific complications. Therefore, DCs are better interpreted as context-dependent regulators of metabolic inflammation and tissue remodeling rather than as a single upstream driver of T2DM. Figure 3 illustrates the potential involvement of DCs in T2DM-associated immunometabolic remodeling.

## 3. Shared Immunometabolic Mechanisms of DCs in Diabetes

**Figure 4:**
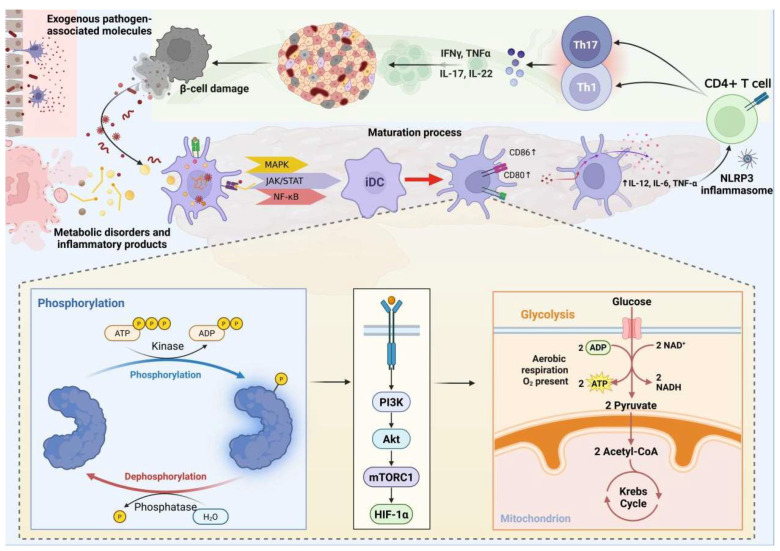
Convergent inflammatory and metabolic signals drive dendritic cell activation and immunometabolic remodeling in diabetes. This schematic illustrates how exogenous pathogen-associated molecules, metabolic disorders, inflammatory products, and β-cell damage-associated signals may promote dendritic cell (DC) activation in diabetes. These stimuli activate intracellular signaling pathways, including MAPK, JAK/STAT, and NF-κB, thereby driving the transition from immature DCs (iDCs) to mature DCs. Mature DCs upregulate co-stimulatory molecules such as CD80 and CD86 and produce inflammatory cytokines, including IL-12, IL-6, and TNF-α. These changes support CD4^+^ T-cell activation and polarization toward Th1 and Th17 responses, which are associated with IFN-γ, TNF-α, IL-17, and IL-22 production. NLRP3 inflammasome activation may further contribute to inflammatory amplification and β-cell injury. The lower panels highlight intracellular regulatory modules involved in DC functional remodeling, including phosphorylation–dephosphorylation events, PI3K–Akt–mTORC1–HIF-1α signaling, and glycolytic/mitochondrial metabolic reprogramming. Together, the figure emphasizes how inflammatory and metabolic cues converge on DC maturation, T-cell polarization, inflammasome activation, and immunometabolic remodeling in diabetes. Abbreviations: Acetyl-CoA, acetyl-coenzyme A; ADP, adenosine diphosphate; Akt, protein kinase B; ATP, adenosine triphosphate; CD, cluster of differentiation; DCs, dendritic cells; HIF-1α, hypoxia-inducible factor-1α; iDCs, immature dendritic cells; IFN, interferon; IL, interleukin; JAK/STAT, Janus kinase/signal transducer and activator of transcription; MAPK, mitogen-activated protein kinase; mTORC1, mechanistic target of rapamycin complex 1; NAD^+^, nicotinamide adenine dinucleotide; NADH, reduced nicotinamide adenine dinucleotide; NF-κB, nuclear factor-κB; NLRP3, NLR family pyrin domain-containing protein 3; PI3K, phosphoinositide 3-kinase; Th1, T helper 1 cell; Th17, T helper 17 cell; TNF-α, tumor necrosis factor-α. Created in BioRender. Jin, F. (2026) https://BioRender.com/qedzzbx.

In T1DM and T2DM, DCs operate within distinct pathological contexts; nevertheless, in both diseases they can sense β-cell stress, tissue injury, danger signals, and inflammatory or metabolic stimuli, and translate these inputs into antigen presentation, co-stimulatory signaling, cytokine release, T-cell polarization, and changes in immune tolerance. This section focuses on the shared molecular regulatory mechanisms of DCs in diabetes, including danger signal sensing, metabolic reprogramming, inflammasome activation, cytokine networks, and tolerance-to-inflammation transitions. This framework highlights partial convergence at the level of DC function rather than immunopathological equivalence between the two diseases. A conceptual overview of how inflammatory and metabolic cues converge on DC maturation, T-cell polarization, inflammasome activation, and metabolic reprogramming in diabetes is shown in Figure 4.

### 3.1. Shared DC Dysfunction and Divergent Disease Outcomes in Diabetes

Although T1DM and T2DM expose DCs to different upstream triggers, the major point of convergence is the dysfunctional DC state induced by persistent danger sensing and metabolic stress. In T1DM, DC-activating inputs mainly arise from virus-associated PAMPs, β-cell autoantigens, and DAMPs released by injured β-cells, whereas in T2DM they are more commonly derived from hyperglycemia, free fatty acids, oxidized lipids, AGEs, adipokines, and gut-derived inflammatory signals [77,78,79,80,81,82]. These heterogeneous inputs can engage partially overlapping DC sensing systems, including TLRs, RAGE, CD36, LOX-1, and P2X7, and activate downstream pathways such as NF-κB, MAPK, IRFs, JAK–STAT, and PI3K–Akt–mTOR signaling [83,84,85,86]. The shared consequence is a shift in DCs away from homeostatic or tolerogenic regulation toward an immunogenic and metabolically activated state, characterized by increased co-stimulatory molecule expression, altered antigen-presenting activity, enhanced pro-inflammatory cytokine production, and weakened capacity to maintain peripheral immune tolerance.

This shared DC dysfunction can generate overlapping inflammatory outputs in both forms of diabetes, including cytokine amplification, enhanced T-cell polarization, impaired immune regulation, and persistent tissue inflammation. However, these common abnormalities do not lead to identical disease outcomes. In T1DM, the presence of β-cell autoantigens and a permissive autoimmune background allows activated DCs to promote β-cell antigen presentation, cross-priming, Th1/Th17 polarization, CD8^+^ T-cell activation, and amplification of insulitis [45,87]. In T2DM, similar DC activation programs are driven mainly by chronic nutrient excess and metabolic stress; therefore, they more often contribute to adipose tissue inflammation, hepatic and vascular immune remodeling, β-cell stress, insulin resistance, and complication-associated inflammation rather than classical β-cell autoimmunity [14,73]. Thus, shared downstream signaling modules can produce common inflammatory outputs while giving rise to divergent pathological outcomes.

The functional consequences of danger sensing are further shaped by DC metabolic reprogramming. Steady-state or tolerogenic DCs generally rely more on oxidative phosphorylation and fatty acid oxidation to maintain a low inflammatory tone and tolerance-inducing capacity. In contrast, immunogenic DC activation is accompanied by enhanced glycolysis, activation of mTOR and hypoxia-inducible factor-1α (HIF-1α), mitochondrial remodeling, and changes in lipid metabolism [88]. These metabolic changes support the energetic and biosynthetic demands of DC maturation, migration, antigen processing, and cytokine production. In the diabetic environment, PI3K–Akt–mTOR–HIF-1α-related metabolic signaling may support the maintenance of immunogenic DC functions by linking danger or metabolic sensing to glycolytic remodeling, co-stimulatory molecule expression, cytokine production, and antigen-presenting activity [41,89]. In T1DM, this may facilitate β-cell antigen presentation in an autoimmune context, whereas in T2DM it may enhance DC responsiveness to hyperglycemia, lipids, AGEs, and inflammatory cytokines, thereby contributing to chronic metabolic inflammation.

Metabolic reprogramming may also prolong DC functional changes through epigenetic mechanisms. Metabolites such as acetyl-coenzyme A, α-ketoglutarate, succinate, and nicotinamide adenine dinucleotide (NAD^+^) can serve as substrates or cofactors for chromatin-modifying enzymes, thereby influencing histone acetylation, methylation, and chromatin accessibility [90]. Thus, long-term hyperglycemia, lipotoxicity, or chronic inflammation may stabilize inflammatory gene expression programs in DCs. However, direct evidence that DCs acquire a stable trained-immunity or metabolic-memory state in diabetes remains insufficient and requires further validation using human samples, DC subset-specific analyses, and functional assays.

### 3.2. Inflammasomes and Cytokine Networks Amplify Immunometabolic Imbalance

The NLR family pyrin domain-containing protein 3 (NLRP3) inflammasome is a key inflammatory platform linking metabolic stress, cellular damage, and inflammatory output. ATP, saturated fatty acids, reactive oxygen species (ROS), cholesterol crystals, uric acid crystals, and glycoxidation products can all contribute to NLRP3 inflammasome priming or activation, thereby promoting caspase-1-dependent maturation and release of IL-1β and IL-18 [30,91,92]. In the diabetic milieu, the NLRP3–IL-1β axis may affect β-cell function, T-cell polarization, and the persistence of tissue inflammation. IL-1β can induce endoplasmic reticulum stress, oxidative stress, β-cell dysfunction, and cellular injury, and can synergize with factors such as IL-6 and IL-23 to promote Th17 responses or suppress regulatory immunity [93,94]. In T1DM, this process may aggravate autoimmune insulitis; in T2DM, it is more deeply embedded within inflammatory networks driven by lipotoxicity, glucotoxicity, macrophage activation, and β-cell metabolic stress. Importantly, the NLRP3–IL-1β axis is not DC-specific, as macrophages, monocytes, and other antigen-presenting cells also contribute to this pathway. Therefore, DCs should be regarded as integral components of this inflammatory amplification network, rather than as its sole source or independent driver. In addition to IL-1β, DC-derived IL-12, IL-6, IL-23, TNF-α, and IFN-I can also influence immune balance in diabetes. IL-12 promotes Th1 differentiation and IFN-γ production; IL-6, IL-1β, and IL-23 contribute to the induction, expansion, or maintenance of Th17 responses; and IFN-I may enhance β-cell immunogenicity or promote metabolic tissue inflammation. The effects of these cytokines depend on disease type, tissue site, DC subset, and inflammatory stage. Therefore, DC-mediated cytokine networks should be viewed as dynamic regulatory systems rather than as fixed, unidirectional pro-inflammatory programs.

### 3.3. Tolerance-to-Inflammation Switching and Disease Chronicity

One of the core functions of DCs is to determine whether antigen presentation leads to immune tolerance or effector activation. Under steady-state conditions, immature or tolerogenic DCs (tolDCs) can maintain peripheral immune tolerance through antigen presentation with minimal co-stimulation, immunosuppressive cues, and Treg induction. In contrast, when DCs are exposed to PAMPs, DAMPs, or metabolic stress signals, they upregulate MHC molecules, CD80, CD86, CD40, and pro-inflammatory cytokines, shifting antigen presentation toward an immunogenic response.

This functional conversion has distinct pathological implications in T1DM and T2DM. In T1DM, immunogenic DC conversion can promote β-cell autoantigen presentation and autoreactive T-cell expansion, thereby driving insulitis and antigen spreading. In T2DM, DC functional conversion usually does not manifest as classical β-cell autoimmunity but instead contributes to immunometabolic feedback loops by shaping immune environments in adipose tissue, the liver, the vasculature, and the islet microenvironment. Hyperglycemia, lipotoxicity, and inflammatory cytokines can enhance DC activation, whereas DC-mediated inflammation and T-cell responses may further aggravate insulin resistance and β-cell stress.

Overall, the common relevance of DCs in T1DM and T2DM does not lie in initiating the same disease processes, but in their ability to transform tissue injury and metabolic pressure into sustained immune responses through coordinated danger signal sensing, metabolic reprogramming, inflammasome activation, cytokine output, antigen presentation, and T-cell regulation. In T1DM, this process is mainly reflected in β-cell autoantigen presentation, autoreactive T-cell priming, and amplification of insulitis. In T2DM, it is more evident in immunometabolic remodeling of adipose tissue, the liver, the vasculature, and the islet microenvironment. Therefore, DCs are better understood as immunometabolic regulatory nodes in diabetes rather than as a shared central cause of both diseases.

## 4. Therapeutic Reprogramming of DC Molecular States

Building on the mechanisms discussed above, DC-related therapeutic strategies should aim not to eliminate DCs or broadly suppress their function, but to reprogram their dysregulated functional states. Ideally, DC modulation should preserve basal immune surveillance while reducing aberrant inflammatory output, restoring antigen-specific immune tolerance, and limiting chronic immunometabolic inflammation. Current evidence supports several translationally relevant directions, including modulation of metabolic and inflammatory pathways, induction of antigen-specific tolerance, engineering of delivery platforms, and patient-stratified clinical application. Representative preclinical strategies targeting DC metabolic state, inflammatory output, antigen-specific tolerance, and delivery-based reprogramming are summarized in Table 1.

### 4.1. Modulating DC Metabolic and Inflammatory Pathways

The immunogenic activation of DCs is closely linked to their metabolic state. Pathways such as AMP-activated protein kinase (AMPK), mTOR, HIF-1α, NF-κB, MAPK, and the NLRP3 inflammasome connect metabolic stress to immune output and represent important therapeutic targets for DC functional reprogramming. AMPK activation is generally associated with metabolic homeostasis and anti-inflammatory states, whereas activation of the mTOR–HIF-1α axis can promote enhanced glycolysis, co-stimulatory molecule expression, and pro-inflammatory cytokine production. Therefore, modulation of the AMPK–mTOR metabolic axis may help reduce immunogenic DC activation and inflammatory output [95,96].

Some metabolic drugs can indirectly regulate DC function. Metformin can modulate mTOR/STAT3 and related signaling through AMPK-dependent mechanisms, thereby reducing DC activation, pro-inflammatory cytokine secretion, and T-cell stimulatory capacity [95,96]. The mTOR inhibitor rapamycin can suppress mTOR-related metabolic programs and reduce immunogenic DC activation, thereby promoting a more tolerogenic functional state [41]. Peroxisome proliferator-activated receptor-γ (PPAR-γ) agonists may also affect DC maturation, migration, and T-cell stimulatory function by regulating lipid metabolism, MAPK, and NF-κB signaling [97,98,99,120]. However, these drugs usually act simultaneously on the liver, adipose tissue, muscle, macrophages, T cells, and β-cells. Therefore, they should be viewed as systemic metabolic or anti-inflammatory interventions with DC-related immunomodulatory effects rather than as strictly DC-targeted therapies.

The NLRP3 inflammasome is also an important candidate target for DC functional reprogramming. Inhibition of the NLRP3–caspase-1–IL-1β axis may reduce the pro-inflammatory output of DCs and other antigen-presenting cells and affect β-cell stress, Th17-related responses, and metabolic tissue inflammation [30,91,92]. However, because NLRP3 is not specific to DCs, interventions targeting this axis should be regarded as broader modulation of innate immune inflammatory networks rather than strictly DC-targeted therapy. Some natural products or bioactive small molecules, such as dioscin and the aqueous extract of Passiflora alata leaves (LAEPAL), may also regulate DC inflammatory states or tolerogenic phenotypes, but their in vivo specificity and clinical relevance require further validation [100,101].

### 4.2. Rebuilding Antigen-Specific Immune Tolerance

T1DM provides a particularly relevant setting for the development of DC-mediated antigen-specific tolerance therapy. Its key immunopathological features include β-cell autoantigen presentation, autoreactive T-cell activation, and breakdown of immune tolerance. Therefore, reprogramming DCs from immunogenic antigen-presenting cells into tolDCs represents an important strategy for restoring β-cell antigen-specific tolerance. TolDCs typically exhibit low levels of co-stimulatory molecules, enhanced immunosuppressive signaling, and the capacity to induce Tregs and type 1 regulatory T cells (Tr1), with features such as reduced CD80/CD86/CD40 expression and upregulation of tolerance-related molecules, including programmed cell death ligand 1 (PD-L1), immunoglobulin-like transcript 3 (ILT3), indoleamine 2,3-dioxygenase-1 (IDO-1), IL-10, and transforming growth factor-β (TGF-β) [104,105,121].

tolDC vaccines are among the most representative strategies. Their basic workflow usually involves the generation of DCs from patient-derived peripheral blood monocytes, induction of a tolerogenic phenotype using vitamin D3, IL-10, TGF-β, dexamethasone, rapamycin, or other immunomodulatory agents, and antigen loading with insulin, proinsulin, GAD65, or other β-cell-related antigens before reinfusion [104,105,121]. The aim of this strategy is not broad immunosuppression but the suppression of autoreactive T-cell responses and enhancement of regulatory immunity in a β-cell antigen-specific context. Preclinical studies have shown that tolDCs can suppress effector T-cell activation, promote Treg expansion, reduce insulitis, and delay diabetes onset [106]. In vitro studies further suggest that insulin-loaded tolDCs can induce insulin-specific hyporesponsiveness in CD4^+^ T cells, suppress T-cell proliferation, and modulate cytokine balance [104]. Early clinical studies support the favorable safety profile and immunomodulatory effects of tolDC therapy. Autologous DC/tolDC vaccines pulsed with proinsulin peptides or Hsp60-derived peptides have generally been well tolerated in patients with T1DM and have been associated with reductions in selected antigen-specific autoreactive T-cell responses. Some studies have also reported slower C-peptide decline or changes in regulatory B-cell and T-cell populations [107,122,123,124,125]. However, current evidence mainly supports safety and immunological biomarker changes, whereas evidence for long-term preservation of β-cell function, improvement in glycated hemoglobin (HbA1c), reduction in insulin requirements, and durable clinical benefit remains insufficient. Future studies should further optimize antigen selection, timing of administration, patient stratification, and efficacy-monitoring endpoints.

### 4.3. Engineered DCs and Precision Delivery Platforms

Engineering approaches can improve the precision and controllability of DC reprogramming. Through genetic engineering or molecular modification, DCs can be designed to express immunoregulatory molecules, such as inhibitory ligands, IL-10, heme oxygenase-1 (HO-1), autoimmune regulator (Aire), or β-cell-related antigens, thereby enhancing antigen-specific immunoregulatory capacity [108,109,110,111]. For example, DCs co-expressing self-antigens and IL-10 can deliver immunosuppressive signals during antigen presentation and promote Tr1- or Treg-related responses [109]. HO-1- or Aire-related strategies may limit autoimmune responses by reducing inflammatory maturation or enhancing tolerance to self-antigens [110,111]. However, engineered DCs still face challenges related to the safety of genetic modification, in vivo persistence, phenotypic stability, migration control, and manufacturing standardization.

Biomaterial delivery systems provide more precise spatiotemporal control over DC reprogramming. Nanoparticles, liposomes, poly(lactic-co-glycolic acid) (PLGA) microparticles, hydrogels, microneedles, and mucosal delivery systems can co-deliver β-cell antigens with vitamin D3, TGF-β, granulocyte-macrophage colony-stimulating factor (GM-CSF), rapamycin, or other immunomodulatory molecules to DCs, thereby creating a non-inflammatory antigen-presenting environment and promoting Treg induction and the establishment of antigen-specific tolerance [102,112,113,114,115,116,117,118,119]. These platforms may be particularly suitable for early-stage or high-risk T1DM, but issues including delivery targeting, dose control, material safety, and the long-term stability of tolerance still need to be addressed. Extracellular vesicles represent a promising cell-free strategy for DC modulation. In a diabetic wound model, exosomes derived from TNF-α-primed adipose-derived stem cells (ADSCs) inhibited DC overactivation through the miR-146a-5p/TXNIP/NLRP3 axis and promoted local inflammation resolution and tissue repair [103]. These findings suggest that exosomes could serve as potential tools for regulating DC inflammatory states and local tissue repair, although their targeting specificity, quality control, dose standardization, and long-term safety require further validation.

### 4.4. Clinical Translation and Future Challenges

DC reprogramming strategies have moved from preclinical development into early clinical exploration. The strategy closest to clinical translation is the induction of antigen-specific tolerance in T1DM [122,123,124,125,126]. Autologous DC/tolDC vaccines, antigen-loaded DCs, and some systemic immunomodulatory strategies have generally shown a favorable safety profile and evidence of immunomodulatory effects in early studies. These approaches have been associated with reductions in selected antigen-specific autoreactive T-cell responses, changes in Treg and Regulatory B cell (Breg) populations, and signals of C-peptide preservation (Table 2). However, existing studies are limited by small sample sizes, short follow-up periods, and heterogeneity in antigen selection, DC preparation methods, routes of administration, and endpoint design. Thus, whether DC-related therapies can durably preserve β-cell function, reduce insulin requirements, and improve metabolic outcomes remains to be determined in larger, standardized randomized controlled trials.

In T2DM, available clinical evidence for DC-related interventions remains limited to exploratory studies in diabetes-associated complications rather than glycemic control or antigen-specific tolerance induction [127,128] (Table 2). Unlike T1DM tolDC strategies aimed at restoring β-cell antigen-specific immune tolerance, the clinical studies in T2DM-associated neuropathy and nephropathy used autologous GM-CSF/IL-4-generated DCs without defined antigen loading. Their reported effects, including improvement of neuropathy symptoms, a trend toward increased TGF-β, reduced albuminuria, and suppression of TNF-α, are therefore better interpreted as broad immunomodulatory and anti-inflammatory actions. This distinction is consistent with the non-classical autoimmune nature of most T2DM complications. Future studies should further clarify which patient subgroups, complication stages, and inflammatory phenotypes are most likely to benefit from DC-related immunomodulation.

Further translation of DC-related therapies still faces three key challenges. First, DCs are highly heterogeneous, and different subsets may exert pro-inflammatory, tolerogenic, or compensatory functions in different tissues and disease stages. Therefore, therapeutic strategies should aim for antigen-, subset-, tissue-, and disease-stage-specific functional reprogramming. Second, patients with diabetes are highly heterogeneous in disease duration, residual β-cell function, degree of obesity, insulin resistance level, complication burden, and immune phenotype, indicating the need for patient stratification based on disease stage and immunometabolic features. Finally, reliable human DC functional biomarkers suitable for clinical translation are still lacking. Future studies should integrate human tissue samples, single-cell omics, spatial omics, and functional validation experiments to define the state transitions, tissue localization, and immune outputs of distinct DC subsets and establish biomarker systems for patient selection, efficacy prediction, and immune monitoring.

Overall, DC reprogramming therapy provides a mechanism-guided concept for immunometabolic intervention in diabetes. In T1DM, the primary goal is to rebuild β-cell antigen-specific tolerance. In T2DM, potential is more evident in regulating chronic metabolic inflammation, tissue immune imbalance, and complication-related inflammatory responses. Future progress will require a clear definition of mechanisms of action, target patient populations, immune endpoints, and metabolic endpoints before DC-related strategies can move from experimental immunomodulation toward precision immunometabolic intervention.

## 5. Conclusions and Perspectives

DCs play critical, yet context-dependent, roles in diabetic immunopathology. In T1DM, DCs mainly participate in tolerance breakdown, amplification of insulitis, and the persistence of autoimmune responses through β-cell autoantigen presentation, danger signal sensing, enhanced co-stimulation, and T-cell polarization. In T2DM, DCs more often act as modulators of metabolic inflammation and tissue immune remodeling, contributing to immunometabolic regulation within adipose tissue, the liver, the vasculature, and pancreatic islets in the context of obesity, insulin resistance, dyslipidemia, and chronic low-grade inflammation. Therefore, DCs are better viewed as immunometabolic regulatory nodes in diabetes rather than as isolated contributors to a single pathogenic pathway.

Mechanistically, diabetes-associated PAMPs, DAMPs, free fatty acids, AGEs, oxidized lipids, and inflammatory cytokines can alter DC functional states through receptors such as TLRs, RAGE, CD36, LOX-1, and P2X7 and their downstream pathways, further influencing metabolic reprogramming, inflammasome activation, antigen presentation, cytokine output, and the balance between tolerance and inflammation. These processes provide a molecular basis for understanding the partial convergence between T1DM autoimmunity and T2DM metabolic inflammation. Therapeutically, reprogramming DC molecular states has important translational potential. tolDC vaccines, antigen-specific delivery systems, engineered DCs, modulation of metabolic and inflammatory pathways, and extracellular vesicles can regulate DC antigen presentation, inflammatory output, and immune tolerance at different levels, providing new directions for immunometabolic intervention in diabetes. At present, clinical evidence mainly supports a favorable safety profile and immunomodulatory effects, whereas long-term disease-modifying benefits remain to be further validated.

Future studies should integrate human tissue samples, single-cell omics, spatial omics, and functional assays to define the precise functional roles of DC subsets across different disease stages and tissue environments and to establish DC functional biomarkers for patient stratification and therapeutic monitoring. Only by establishing clearly defined disease contexts, mechanisms of action, and target patient populations can DC-targeted strategies be developed into precision immunometabolic interventions.

## Figures and Tables

**Table 1:** Representative preclinical studies of DC-related therapeutic reprogramming in diabetes.

Therapeutic Direction	Disease Context/Model	Experimental System	DC-Related Intervention	Main Preclinical Findings	Mechanistic Interpretation and Limitations	Refs.
Metabolic pathway modulation: metformin	T1DM; autoimmune diabetes models and DC-focused mechanistic studies	In vivo models and DC functional assays	Metformin-mediated modulation of mTOR/STAT3 suppression and DC metabolic reprogramming	Reduced DC activation and pro-inflammatory output; promoted tolDC features; associated with reduced effector T-cell responses and increased regulatory immunity in models.	Systemic metabolic/immunomodulatory intervention with DC-related effects; not a DC-specific targeted therapy.	[95,96]
PPAR-γ pathway modulation	Diabetes-relevant metabolic inflammation	Human or experimental DC systems	PPAR-γ agonist-mediated regulation of lipid metabolism, MAPK and NF-κB signaling in DCs	Reduced TLR-mediated activation, altered DC immunogenicity, and modulation of maturation, migration, or T-cell stimulatory capacity.	Supports a DC-related mechanism for systemic metabolic drugs; evidence is mostly mechanistic and not diabetes-specific in all studies.	[97,98,99]
Natural product/small-molecule modulation: LAEPAL	T1DM-relevant NOD immune context	In vitro NOD bone marrow-derived APCs and T cells	Aqueous extract of Passiflora alata leaves (LAEPAL) to condition APCs including DCs	Reduced CD86 and effector T-cell proliferation; increased IDO-related tolerogenic features and Treg polarization.	Represents tolerogenic APC conditioning rather than strictly DC-specific therapy; requires in vivo and clinical validation.	[100]
Natural product/small-molecule modulation: dioscin	T2DM-like high-glucose inflammatory environment	In vitro DCs under high-glucose and oxLDL-related stress	Dioscin treatment	Reduced oxLDL uptake, scavenger receptor expression, ROS, IL-6/IL-12 and inflammatory activation; increased anti-inflammatory tendency.	Likely acts through p38 MAPK inhibition; preclinical in vitro evidence only.	[101]
Curcumin-loaded liposomes targeting inflammatory DCs	Obesity-associated insulin resistance/T2DM-like metabolic inflammation	Obese mouse model with hepatic inflammatory DCs and adipose tissue inflammation	Curcusomes targeting inflammatory DCs/macrophages	Improved glucose tolerance and insulin resistance-related parameters; reduced pro-inflammatory cytokines and inflammatory DC/macrophage activity.	Acts on inflammatory DCs and macrophages through NF-κB inhibition; not strictly DC-specific.	[102]
Extracellular vesicle-based DC modulation	T2DM-associated wound inflammation	Diabetic wound model and high-glucose DC experiments	TNF-α-primed ADSC-derived exosomes	Suppressed DC overactivation and NLRP3 inflammasome activity; promoted inflammatory resolution, angiogenesis, collagen deposition and wound repair.	miR-146a-5p/TXNIP/NLRP3 axis mediates DC inflammatory repression; cell-free but not strictly DC-specific.	[103]
tolDC induction with insulin antigen	T1DM antigen-specific tolerance	Human DCs and T cells from individuals with T1DM	IL-10/TGF-β-treated DCs pulsed with insulin	Induced insulin-specific hyporesponsiveness in CD4^+^ effector/memory T cells and modulated cytokine balance.	Supports β-cell antigen-specific tolerance induction by tolDCs; in vitro patient-cell evidence.	[104]
Vitamin D3-induced tolDCs	T1DM prevention/tolerance induction	Human therapeutic tolDC manufacturing and NOD mouse studies	1,25-dihydroxyvitamin D3-induced tolDCs	Generated stable and reproducible tolDCs; in NOD mice, promoted tolDCs with migratory properties relevant to therapy.	Supports manufacturable tolDC platforms; antigen choice and in vivo stability remain important issues.	[105,106]
Islet antigen vaccine with cyclosporin A	T1DM prevention model	Murine autoimmune diabetes model	Islet-derived multipeptides combined with cyclosporin A	Prevented diabetes in mice and induced antigen-specific peripheral Tregs while suppressing autoreactive responses.	Likely involves tolerogenic antigen presentation and pTreg induction; broader vaccine strategy rather than pure DC therapy.	[107]
Checkpoint-engineered DCs	T1DM autoimmunity	Engineered DC-based preclinical diabetes model	DCs engineered to activate T-cell checkpoint pathways during β-cell antigen presentation	Promoted protection from T1DM and reduced pathogenic T-cell responses.	Couples β-cell antigen presentation with inhibitory checkpoint signaling to favor tolerance.	[108]
IL-10-engineered DC platform	Antigen-specific tolerance strategy	Engineered DC-based preclinical platform	DCs engineered to express IL-10 during antigen presentation	Enhanced antigen-specific regulatory responses and reduced effector T-cell activation in T cell-mediated disease settings.	Relevant platform for diabetes tolerance strategies, but diabetes-specific evidence should be interpreted cautiously.	[109]
Aire-overexpressing DCs	T1DM onset delay	Preclinical T1DM mouse model preclinical diabetes model	Transplantation of Aire-overexpressing bone marrow-derived DCs	Delayed diabetes onset, reduced autoimmune features, preserved islet structure, and shifted T-cell balance toward regulation.	Aire expression may enhance self-antigen tolerance and reduce autoreactive T-cell responses.	[110]
HO-1 restoration in DCs	T1DM prevention/reversal models	NOD mouse model	Genetic restoration or induction of HO-1 expression in DCs	Protected from T1DM, reduced insulitis, delayed disease onset, and stabilized glycemia in models.	HO-1 promotes a more tolerogenic and anti-inflammatory DC state; genetic strategy requires safety evaluation.	[111]
Oral antigen delivery to intestinal DCs	T1DM oral tolerance	NOD mouse model	Targeted delivery of antigen to intestinal DCs	Induced oral tolerance and prevented autoimmune diabetes in NOD mice.	Promotes gut-associated tolerogenic antigen presentation and regulatory immunity.	[112]
Dual-sized microparticle systems	T1DM prevention and recent-onset intervention	NOD mouse models	PLGA microparticles delivering GM-CSF, TGF-β1, vitamin D3 and insulin autoantigen to modulate DCs	Generated suppressive/tolDC phenotypes, increased Tregs and PD-1 expression, prevented or delayed diabetes in preclinical models; combination with anti-CD3 did not improve late-stage prevention.	Local DC conditioning can promote antigen-specific tolerance; efficacy may depend on disease stage and combination strategy.	[113,114,115]
Hydrogel-based DC/tolDC modulation	T1DM antigen-specific tolerance	Mouse autoimmune diabetes model and antigen-specific hydrogel platform studies	Injectable hydrogels or tolDC-loaded immunomodulatory hydrogels delivering antigens or tolerogenic cues	Promoted local Treg enrichment or delayed diabetes onset without accelerating disease.	Biomaterials may provide localized non-inflammatory antigen presentation and improve tolDC activity.	[116,117]
Autoantigen-decorated microparticle formulation	Mid-stage insulitic autoimmunity in T1DM	NOD mouse model	All-trans retinoic acid and TGF-β1 single microparticle formulation decorated with autoantigen	Arrested progression at mid-stage insulitic autoimmunity and reduced insulitis.	Combines antigen and tolerogenic cues to promote antigen-specific immune modulation.	[118]
Mannosylated alginate nanoparticle delivery	T1DM antigen delivery	NOD mouse model	Mannosylated alginate nanoparticles loaded with insulin peptide	Delayed but did not prevent T1DM onset; increased regulatory responses but also risked pathogenic cross-presentation.	Illustrates the need to control antigen routing and avoid MHC I-mediated diabetogenic CD8+ T-cell activation.	[119]

Abbreviations: Aire, autoimmune regulator; APCs, antigen-presenting cells; DCs, dendritic cells; GM-CSF, granulocyte-macrophage colony-stimulating factor; HO-1, heme oxygenase-1; IDO, indoleamine 2,3-dioxygenase; IL, interleukin; LAEPAL, aqueous extract of Passiflora alata leaves; MAPK, mitogen-activated protein kinase; MHC, major histocompatibility complex; miR, microRNA; mTOR, mammalian target of rapamycin; NF-κB, nuclear factor-κB; NLRP3, NLR family pyrin domain-containing protein 3; NOD, non-obese diabetic; oxLDL, oxidized low-density lipoprotein; PD-1, programmed cell death protein 1; PLGA, poly(lactic-co-glycolic acid); PPAR-γ, peroxisome proliferator-activated receptor-γ; ROS, reactive oxygen species; STAT3, signal transducer and activator of transcription 3; T1DM, type 1 diabetes mellitus; T2DM, type 2 diabetes mellitus; TGF-β, transforming growth factor-β; TLR, Toll-like receptor; TNF-α, tumor necrosis factor-α; tolDCs, tolerogenic dendritic cells; Tregs, regulatory T cells; TXNIP, thioredoxin-interacting protein.

**Table 2:** Clinical studies of DC-based or DC-related therapeutic interventions in diabetes mellitus.

Study	Experiment Type	Experimental Subjects	Phase	Follow-Up	Enrollment	DC-Based or DC-Related Interventions	Experimental Results	Possible Mechanism	Refs.
Clinical trial (NCT03895996)	Randomized, Double-blind, Placebo-controlled Trial	T1DM patients	1/2	2 years	25	AVT001: Autologous DC vaccine primed with the Hsp60sp peptide.	Safe with no SAEs; associated with significantly less decline in C-peptide AUC compared to placebo at days 150 and 360. No clear differences in HbA1c or insulin dose were observed.	Correcting the defective Q/E CD8+ Treg cell pathway to preserve beta-cell function.	[125]
Clinical trial (2013-005476-18)	Placebo-controlled, dose-escalation trial	T1DM patients	1	3 years	9	Intradermal injection of autologous tolDCs pulsed with a proinsulin peptide (C19-A3).	The therapy was safe and induced a profound, durable reduction in vaccine-specific autoimmune T-cell responses. Some patients showed improved long-term glycemic control (lower HbA1c).	Induction of antigen-specific immune tolerance by modulating T-cell responses: increasing Tregs and reducing proinsulin-reactive effector T cells.	[122,123]
Clinical trial (NCT00445913)	Randomized, double-blind study	T1DM patients	1	1 year	10	Intradermal injection of autologous DCs (some unmanipulated, others engineered ex vivo to an immunosuppressive/tolerogenic state).	The therapy was safe and well-tolerated with no adverse events. A significant increase in peripheral B220+ CD11c- B cells was observed, particularly in recipients of engineered DCs.	The increase in a specific B-cell population (potentially Bregs) may contribute to beneficial immunomodulation in T1DM autoimmunity.	[124]
Clinical trial (NR)	Open-label trial	T1DM patients	1	2 years	12	Weekly intravenous infusions of α1-antitrypsin, aimed at modulating the function of mDCs.	α1-antitrypsin treatment downregulated TLR-induced IL-1β responses in monocytes and mDCs. In a subset of patients (5/12), it was associated with preserved or improved C-peptide levels.	The anti-inflammatory drugα1-antitrypsin specifically targets and suppresses pro-inflammatory (IL-1β) responses in innate immune cells, including DCs, potentially preserving β-cell function.	[126]
Clinical trial (NR)	Open-label, quasi-experimental clinical trial	Type 2 diabetic neuropathy patients	NR	4 weeks	28	Subcutaneous injection of autologous DCs derived from the patient’s own peripheral blood monocytes (cultured with GM-CSF and IL-4).	Significant improvement in neuropathy symptoms. Biomarkers showed a non-significant trend: TGF-β increased, VCAM-1 slightly increased. TGF-β levels correlated negatively with TCNS.	Autologous DCs may exert an anti-inflammatory and immunomodulatory effect, potentially by promoting an anti-inflammatory environment (e.g., via TGF-β) and modulating immune responses to support nerve repair and reduce inflammation in diabetic neuropathy.	[127]
Clinical trial (NR)	Open-label, quasi-experimental clinical trial	Type 2 diabetic nephropathy patients	NR	4 weeks	69	Subcutaneous injection of autologous DCs generated ex vivo from patient PBMCs (cultured with GM-CSF and IL-4).	Significantly reduced albuminuria (UACR decreased and remained lower for 4 weeks). TNF-α levels significantly decreased. No significant changes in eGFR, IL-6, or IL-10.	DC therapy may alleviate albuminuria primarily through an anti-inflammatory effect mediated by suppressing the pro-inflammatory cytokine TNF-α, rather than by enhancing the anti-inflammatory cytokine IL-10.	[128]

Abbreviations: DCs, dendritic cells; T1DM, type 1 diabetes mellitus; tolDCs, tolerogenic dendritic cells; Tregs, regulatory T cells; Bregs, regulatory B cells; mDCs, myeloid dendritic cells.

## Data Availability

No new data were created or analyzed in this study. Data sharing is not applicable to this article.

## Supplementary Materials

The following supporting information can be downloaded at: https://www.mdpi.com/article/10.3390/ijms27136057/s1.

## Abbreviations

The following abbreviations are used in this manuscript:

ADSCs	Adipose-derived stem cells
AGEs	Advanced glycation end products
Aire	Autoimmune regulator
Akt	Protein kinase B
AMPK	AMP-activated protein kinase
APCs	Antigen-presenting cells
APOB	Apolipoprotein B
ATP	Adenosine triphosphate
AUC	Area under the curve
Bregs	Regulatory B cells
CCL2	C-C motif chemokine ligand 2
CCR7	C-C chemokine receptor 7
CD	Cluster of differentiation
cDC1s	Conventional type 1 dendritic cells
cDC2s	Conventional type 2 dendritic cells
CXCL10	C-X-C motif chemokine ligand 10
DAMPs	Damage-associated molecular patterns
DCs	Dendritic cells
DKD	Diabetic kidney disease
eGFR	Estimated glomerular filtration rate
eNOS	Endothelial nitric oxide synthase
GAD65	Glutamic acid decarboxylase 65
GM-CSF	Granulocyte-macrophage colony-stimulating factor
HbA1c	Glycated hemoglobin
HIF-1α	Hypoxia-inducible factor-1α
HMGB1	High-mobility group box 1
HO-1	Heme oxygenase-1
HSP70	Heat shock protein 70
Hsp60	Heat shock protein 60
Hsp60sp	Heat shock protein 60 signal peptide
IA-2	Insulinoma-associated antigen-2
IDO-1	Indoleamine 2,3-dioxygenase-1
IFN-I	Type I interferon
IFNAR	Interferon-α/β receptor
IFN-γ	Interferon-γ
IL	Interleukin
ILT3	Immunoglobulin-like transcript 3
iNKT cells	Invariant natural killer T cells
IRFs	Interferon regulatory factors
JAK–STAT	Janus kinase–signal transducer and activator of transcription
LAEPAL	Aqueous extract of Passiflora alata leaves
LCs	Langerhans cells
LepRb	Leptin receptor b
LOX-1	Lectin-like oxidized low-density lipoprotein receptor-1
MAPK	Mitogen-activated protein kinase
mDCs	Myeloid dendritic cells
MHC	Major histocompatibility complex
miR	MicroRNA
moDCs	Monocyte-derived dendritic cells
mTOR	Mammalian target of rapamycin
MyD88	Myeloid differentiation primary response 88
NAD^+^	Nicotinamide adenine dinucleotide
NETs	Neutrophil extracellular traps
NF-κB	Nuclear factor-κB
NK cells	Natural killer cells
NKT cells	Natural killer T cells
NLRP3	NLR family pyrin domain-containing protein 3
NOD	Non-obese diabetic
NR	Not reported
oxLDL	Oxidized low-density lipoprotein
P2X7	P2X purinoceptor 7
PAMPs	Pathogen-associated molecular patterns
PBMCs	Peripheral blood mononuclear cells
PD-1	Programmed cell death protein 1
PD-L1	Programmed cell death ligand 1
PI3K	Phosphoinositide 3-kinase
PKCβ1	Protein kinase C β1
PLGA	Poly(lactic-co-glycolic acid)
PPAR-γ	Peroxisome proliferator-activated receptor-γ
pDCs	Plasmacytoid dendritic cells
RAGE	Receptor for advanced glycation end products
ROS	Reactive oxygen species
SAEs	Serious adverse events
STAT3	Signal transducer and activator of transcription 3
STZ	Streptozotocin
T1DM	Type 1 diabetes mellitus
T2DM	Type 2 diabetes mellitus
TCR	T-cell receptor
TCNS	Toronto Clinical Neuropathy Score
TGF-β	Transforming growth factor-β
Th1	T helper 1
Th17	T helper 17
TLR	Toll-like receptor
TNF-α	Tumor necrosis factor-α
tolDCs	Tolerogenic dendritic cells
Tregs	Regulatory T cells
Tr1	Type 1 regulatory T cells
TRIF	Toll/interleukin-1 receptor domain-containing adaptor-inducing interferon-β
TXNIP	Thioredoxin-interacting protein
UACR	Urinary albumin-to-creatinine ratio
VCAM-1	Vascular cell adhesion molecule-1
