# Supplementary material for: Dendritic Cells as Immunometabolic Regulatory Nodes in Diabetes: Molecular Mechanisms and Therapeutic Reprogramming
Source: Int J Mol Sci. 2026 Jul 6;27(13):6057. doi: 10.3390/ijms27136057 (PMC13361900; doi:10.3390/ijms27136057)
Supplement: Supplementary file 1 [file ijms-27-06057-s001.zip › ijms-4357743-supplementary.pdf]

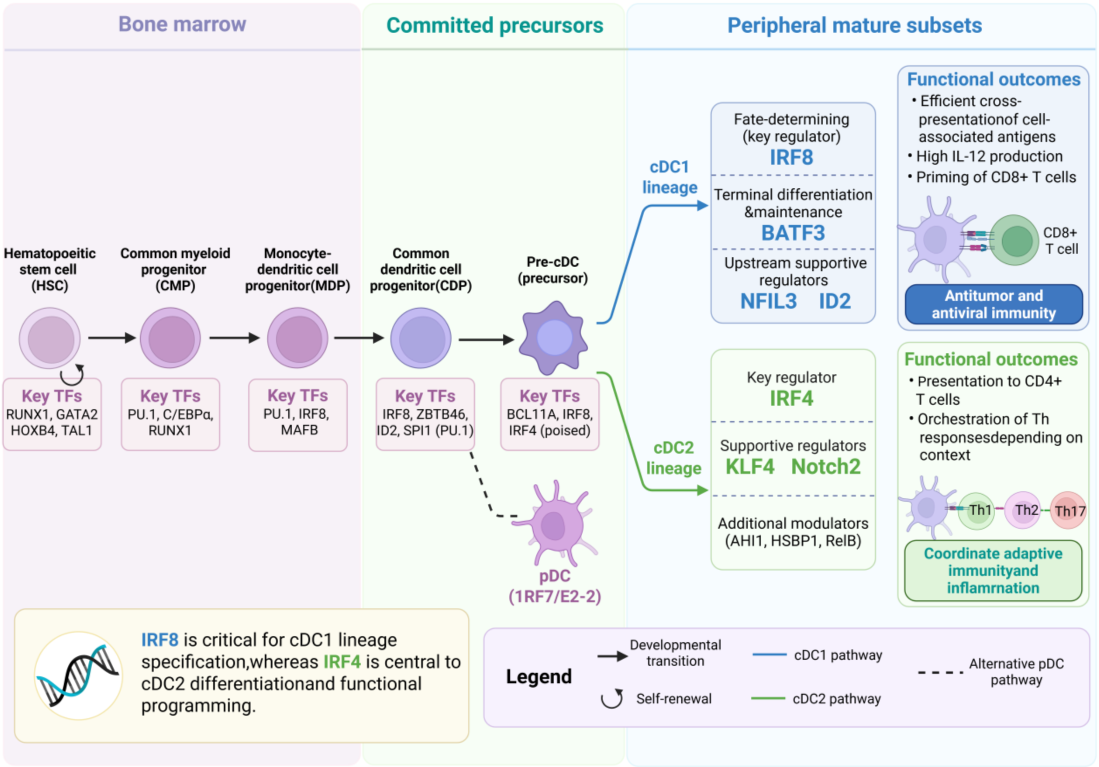

Supplementary Figure S1. Differentiation and transcriptional regulation of cDC1 and cDC2 lineages. This schematic illustrates the developmental trajectory and transcriptional regulation of conventional dendritic cell subsets. Hematopoietic stem cells give rise to common myeloid progenitors, monocyte–dendritic cell progenitors, common dendritic cell progenitors, and pre-cDCs, which subsequently branch into cDC1 and cDC2 lineages. cDC1 development and identity are strongly associated with IRF8, with BATF3 supporting terminal differentiation and maintenance; NFIL3 and ID2 are shown as upstream supportive regulators. Functionally, cDC1s are specialized in efficient cross-presentation, IL-12 production, priming of CD8+ T cells, and antiviral or antitumor immunity. In contrast, cDC2 differentiation and functional programming are closely linked to IRF4, with KLF4 and Notch2 contributing to subset-specific development and function. cDC2s preferentially present antigen to CD4+ T cells and orchestrate context-dependent helper T-cell responses, including Th1, Th2, and Th17 polarization. A related pDC branch is shown in a secondary manner to contextualize DC ontogeny, with E2-2 and IRF7 indicating pDC development and type I interferon-associated function. Abbreviations: AHI1, Abelson helper integration site 1; BATF3, basic leucine zipper ATF-like transcription factor 3; BCL11A, B-cell lymphoma/leukemia 11A; CD, cluster of differentiation; CDP, common dendritic cell progenitor; cDC1, conventional type 1 dendritic cell; cDC2, conventional type 2 dendritic cell; CMP, common myeloid progenitor; DC, dendritic cell; E2-2, transcription factor E2-2; GATA2, GATA binding protein 2; HOXB4, homeobox B4; HSC, hematopoietic stem cell; HSBP1, heat shock factor binding protein 1; ID2, inhibitor of DNA binding 2; IL, interleukin; IRF4, interferon regulatory factor 4; IRF7, interferon regulatory factor 7; IRF8, interferon regulatory factor 8; KLF4, Krüppel-like factor 4; MAFB, MAF bZIP transcription factor B; MDP, monocyte–dendritic cell progenitor; NFIL3, nuclear factor interleukin-3 regulated; Notch2, notch receptor 2; pDC, plasmacytoid dendritic cell; pre-cDC, pre-conventional dendritic cell; PU.1, Spi-1 proto-oncogene; RelB, RELB proto-oncogene, NF-κB subunit; RUNX1, runt-related transcription factor 1; TAL1, T-cell acute lymphocytic leukemia protein 1; TFs, transcription factors; Th1/Th2/Th17, T helper 1/T helper 2/T helper 17 cells. Created in BioRender. Jin, F. (2026) https://BioRender.com/rg4agn3.
